# Valganciclovir as Add-On Therapy Modifies the Frequency of NK and NKT Cell Subpopulations in Disseminated Kaposi Sarcoma Patients

**DOI:** 10.3390/cancers14020412

**Published:** 2022-01-14

**Authors:** Julio Flores-Gonzalez, Lucero A. Ramon-Luing, Ranferi Ocaña-Guzman, Ivette Buendia-Roldan, Beda Islas-Muñoz, Patricia Volkow-Fernández, Leslie Chavez-Galan

**Affiliations:** 1 Laboratory of Integrative Immunology, Instituto Nacional de Enfermedades Respiratorias Ismael Cosio Villegas, Mexico City 14080, Mexico; jfloresg1707@alumno.ipn.mx (J.F.-G.); ramonluing@yahoo.com.mx (L.A.R.-L.); arocana@iner.gob.mx (R.O.-G.); 2 Laboratory of Translational Research in Aging and Pulmonary Fibrosis, Instituto Nacional de Enfermedades Respiratorias Ismael Cosio Villegas, Mexico City 14080, Mexico; ivettebu@yahoo.com.mx; 3 Infectious Diseases Department, Instituto Nacional de Cancerología, Mexico City 14080, Mexico; bedaislas@gmail.com (B.I.-M.); pvolkowf@gmail.com (P.V.-F.)

**Keywords:** valganciclovir, kaposi sarcoma, human herpesvirus 8, HIV, natural killer cells, natural killer T-cells

## Abstract

**Simple Summary:**

Kaposi sarcoma is one disease that develops in people living with HIV with severe immunosuppression and impacts morbidity and associated mortality. This disease is currently treated with antiretroviral therapy and chemotherapy agents that can further contribute to immunosuppression in patients. Thus, searching for new therapies to induce a robust immune system activation in these patients is necessary. Herein, the frequency and phenotype of natural killer subpopulation cells in people living with HIV with Kaposi sarcoma were evaluated. After KS diagnosis, patients started antiretroviral therapy or valganciclovir plus antiretroviral therapy. Results showed that in patients treated with valganciclovir plus antiretroviral therapy, the expression of CD57 and CD27 proteins on natural killer cells was regulated, enhancing the immune response of the study cohort. This finding contributes to understanding more about the immune response of people living with HIV with Kaposi sarcoma.

**Abstract:**

Human herpesvirus-8 infection (HHV-8) is the causative agent of Kaposi sarcoma (KS) and is highly prevalent among people living with HIV (KS/HIV). It has been reported that valganciclovir (VGC) reduces HHV-8 replication in KS/HIV patients. However, currently it is unclear if VGC modifies the frequency and induces changes in markers of immune regulation of immune cells necessary to eliminate HHV8-infected cells, such as Natural Killer (NK) and NK T cells (NKT). This study evaluated the effect of VGC used as antiviral HHV8 therapy in KS patients on the frequency of NK and NKT subpopulations based on the CD27 and CD57 expression, and the immunosenescence markers, PD-1 and KLRG1. Twenty KS/HIV patients were followed-up at baseline (W_0_), 4 (W_4_), and 12 weeks (W_12_) of the study protocol. Among them, 10 patients received a conventional treatment scheme (CT), solely antiretroviral therapy (ART), and 10 patients received a modified treatment regime (MT), including VGC plus ART. In both groups, bleomycin/vincristine was administrated according to the treating physician’s decision. The soluble levels of IL-15, PD-L1, PD-L2, and E-cadherin were quantified across the follow-up. Our results showed that the higher IL-15 levels and lower NK frequencies cells in KS/HIV patients reach almost normal values with both treatments regimes at W_12_. CD27+ NK and NKT cell frequencies increased since W_4_ on KS/HIV patients with MT. Furthermore, PD-1 expression decreased while KLRG1 increased on NK and NKT subpopulations at W_12_, and it is accompanied by increased PD-L1 plasma level since W_4_. Our study highlights the disruption of NK and NKT subpopulations in patients with KS/HIV and explores VGC treatment’s contribution to immune reconstitution during the first weeks of treatment.

## 1. Introduction

People living with HIV (PLWH) are more vulnerable to opportunistic infections (OIs). For instance, the co-infection with sarcoma-associated herpesvirus (KSHV), also called human herpesvirus-8 (HHV-8), is the causative agent of Kaposi Sarcoma, the first malignancy HIV associated and described in the early years of the HIV/AIDS epidemic [1]. Furthermore, before developing combined antiretroviral therapy (cART), over 30% of AIDS patients developed KS [2]; and this disease is highly frequent in men that had sex with men (MSM); in fact, SK was considered a second epidemic among this population [2]. Nowadays, KS incidence has decreased significantly due to cART administering to PLWH; nevertheless, attributable mortality to KS is still high in the first months after cART initiation in KS/HIV patients [3].

Currently, KS is classified in four major epidemiological forms: classic (or Mediterranean) KS (CKS) is a mild or not very aggressive form that is not associated with HIV, affecting older men of Mediterranean, East European, or Jewish heritage; endemic African KS is a more aggressive form than classic KS and has incidence in sub-Saharan Africa among children and adults; iatrogenic immunosuppressive KS, affecting patients receiving chronic immunosuppressive therapy, mostly organ-transplant recipients; and Epidemic KS or AIDS-KS is the most common type of cancer among HIV+ patients, which affects PLHIV [4,5].

KS is considered an atypical tumor associated with immunosuppression [5], and it has been described as a multicentric hyperproliferative disease that most often presents with violaceous skin lesions. These lesions consist of spindle-shaped tumor cells, often accompanied by fibrosis, inflammatory infiltrates, vascular slits, and hemosiderin [6].

KS diagnosis is based on clinical aspects of lesions and pathological features such as whorls of spindle cell with leucocytes infiltration and aberrant proliferation of small vessels that lack basement membrane and leaky behavior, microhemorrhages, and hemosiderin deposits immunohistochemical LANA stain positive. More recently, other complementary studies have been performed, such as HHV-8 viremia [7]. Patients with localized KS generally have a good prognosis with an indolent course without chemotherapy and solely regress with antiretroviral therapy (ART). However, in patients with disseminated KS, the prognosis is adverse, in some cases requiring multiple cycles of chemotherapy and in others with a fulminant course and high mortality within the first month’s ART initiation. In this regard, only the disseminated disease or with lung involvement have been defined as bad prognosis markers [8,9,10,11].

The World Health Organization (WHO) has established the exclusive use of ART in KS/HIV patients; however, epidemiological data shows that KS is an important factor of morbidity and mortality in HIV patients [12]. The mortality attributable to KS occurs in the first months after ART initiation, and it is associated with immune reconstitution inflammatory syndrome (IRIS) [13,14]. New pharmacologic treatments derived from clinical trials have been studied for KSHV infection, including monoclonal antibodies such as pembrolizumab (anti-PD1), chemotherapy with pomalidomide (anti-angiogenic agent) or paclitaxel (antimicrotubular agent), and kinase inhibitors such as selumetinib [7,15]. Valganciclovir (VGC), an oral prodrug of ganciclovir that inhibits viral DNA synthesis, is a standard gold treatment for treating cytomegalovirus end-organ disease and secondary prophylaxis; it has been demonstrated to have an antiviral effect against HHV-8 in vitro, and several studies have proposed the clinical use of VGC as an antiviral to reduce HHV-8 replication in KS/HIV patients [16,17,18].

Natural killer (NK) and natural killer T (NKT) cells are cytotoxic lymphoid cells involved in eliminating infected or transformed cells. The NK cell activation occurs when these cells identify the absence of classical major histocompatibility complex class I (MHC-I) molecules, which provide the inhibitory signals. In this regard, HHV-8 infection impairs the NK cell lytic capacity because it induces down-regulation of MHC-I on infected cells [19]. In contrast, NKT cells are activated through the recognition of glycolipid antigens via T Cell Receptor (TCR) [20].

The interleukin (IL)-15 is a cytokine necessary for the survival, proliferation, metabolism, and functionality of both NK and NKT cells [21]. NK cells are divided into two distinct subsets, one expressing high intensity of CD56 (CD56^high^ NK), which is associated with immunoregulatory functions, and a second expressing low intensity (CD56^dim^ NK), which is associated with high cytotoxic capability [22]. Moreover, the co-expression of CD27 or CD57 defines NK and NKT cells subsets in chronic infections [23,24]. Recently, it was shown that viral infections induce the expression of checkpoint receptors like programmed death-1 (PD-1) and killer cell lectin-like receptor G1 (KLRG1) on NK and NKT cells, regulating the activation of these cells [25,26].

The knowledge about the frequency and phenotype of NK and NKT cell subpopulations during KS/HIV infection is limited; evidence suggests that ART increases the frequency of CD56^bright^CD27^high^ cells and could benefit HIV patients [27]. However, it has not been explored if KS/HIV patients modify their NK and NKT cells profile using ART or ART/VGC treatment scheme.

In this study, we evaluated the frequency and phenotype of NK and NKT cells subpopulations in KS/HIV patients (nested in a clinical trial) divided into two groups, one group treated with a conventional treatment (CT) that included only ART, and a second group treated with intervention treatment (modified treatment, MT), which included the use of VGC plus ART. In both groups, chemotherapy was administrated according to the treating physician. The cells were classified based on the co-expression of CD27, CD57, PD-1, PDL-1, PDL-2, and KLRG1.

## 2. Materials and Methods

### 2.1. Ethics Statement

This study was approved by the Institutional Ethics Committee of the *Instituto Nacional de Enfermedades Respiratorias* (B30–20) and the *Instituto Nacional de Cancerología* (018/085/INI) (CEI/1341/18) both at Mexico City, and the RCT (015/031/INI) (CEI/950/15), which was also registered at NIH Clinical Trails (ID NCT03296553). All participants signed written informed consent. All procedures were performed in agreement with the 1964 Helsinki Declaration and the ethical standards of the Institutional Ethics Committees.

### 2.2. Study Populations

Twenty men >18-year-old with a disseminated KS diagnosis (KS/HIV patients), naïve to ART, were recruited and followed up at *Instituto Nacional de Cancerología*, Mexico City.

**Figure 1:**
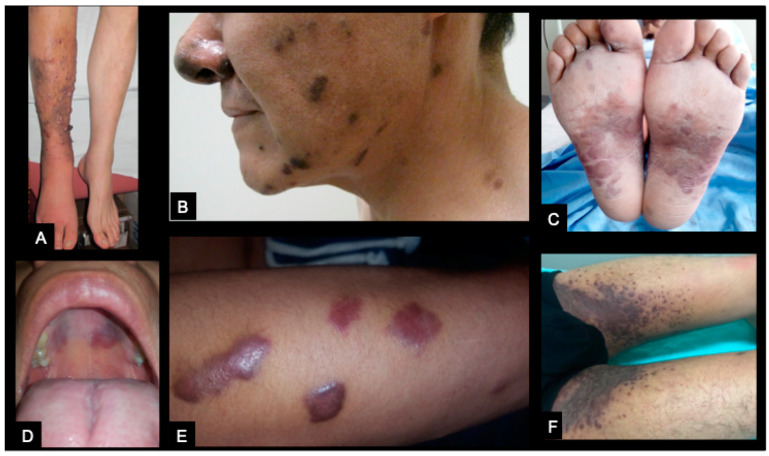
Skin lesions from HIV patients with disseminated Kaposi Sarcoma diagnosis (KS/HIV patients). Representative clinical images showing different clinical presentations of lesions from KS/HIV patients. (**A**) Exophytic and verrucous lesions limited to the right leg with lymphedema. (**B**) Dark plaques in face and nose. (**C**) Erythematous plaques insoles. (**D**) Violaceous macules in the palate. (**E**) Violaceous and elevated plaques in the forearm. (**F**) Bilateral groin infiltration with confluent violaceous plaques.

The disseminated KS diagnosis was defined by at least two of the following criteria: pulmonary, lymph node or gastrointestinal involvement, lymphedema, or ≥30 skin lesions; representative clinical figures of the observed lesions in KS/HIV patients are shown in Figure 1.

Exclusion criteria were patients with any other concomitant malignant disease, Castleman’s disease, active infection with hepatitis B or C virus, cytomegalovirus end-organ disease, steroid treatment for the last 2 months, and/or patients with APACHE score >15 points. Laboratory variables (CD4 and CD8 T-cell counts), moreover cytomegalovirus (CMV), and Epstein-Barr Virus (EBV) were assessed at each visit, and an infectious diseases specialist performed a clinical evaluation. In Table 1 are shown the main clinical characteristics and concomitant coinfections presented by KS/HIV patients during the follow-up times of study. Almost all patients had EBV, while CMV was presented in some patients who resolved the viremia at W_12_. In addition, some KS/HIV patients were diagnosed with concomitant OIs; one patient in the CT group had *Histoplasma capsulatum*, another with *Mycobacterium avium* complex (MAC), and two with *Helicobacter pylori*. One patient with *Histoplasma capsulatum*, another with MAC, and three with *Helicobacter pylori* coinfections were diagnosed in the MT group. Syphilis and neurosyphilis also were presented in some patients. All patients with coinfection received specific treatment for their infectious diagnosis.

**Figure 2:**
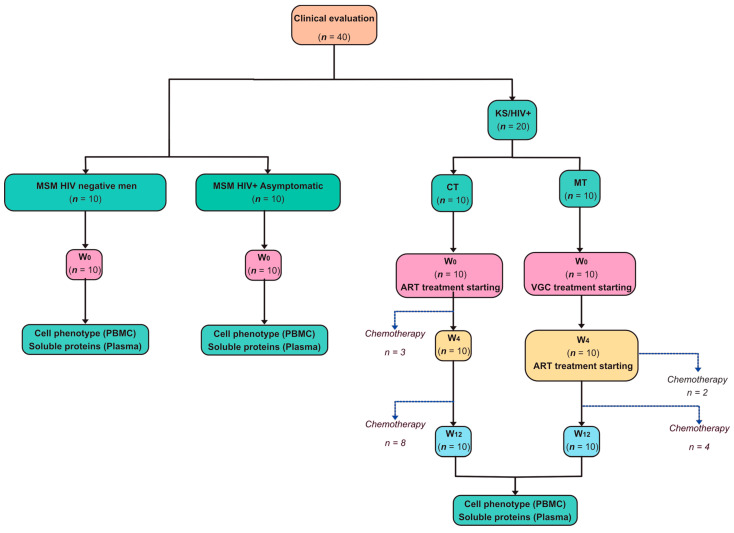
Workflow of KS/HIV patients recruitment and follow-up. A total of 40 participants were enrolled, 20 with KS/HIV diagnostic, 10 HIV+ asymptomatic, and 10 HIV-MSM (Men who have Sex with Men). The KS/HIV group was divided according to the received scheme treatment; 10 KS/HIV patients received the conventional treatment scheme (CT = ART) since baseline (W_0_), and 10 KS/HIV patients received the modified treatment scheme (MT = VGC + ART) starting with VGC at W_0_ and ART at W_4_. The blue line shows the number of patients that received chemotherapy across follow-up. Cell phenotype using peripheral mononuclear cells (PBMC) and soluble proteins in plasma were evaluated on KS/HIV groups at W_0_ and follow-up at W_4_ and W_12_. In addition, the HIV+ asymptomatic men and HIV negative MSM groups were evaluated at W_0_. The number of samples processed per group for each experimental protocol is shown in parentheses.

It was an open-label parallel-group randomized trial divided into two groups (*n* = 10/group), in agreement with the received scheme treatment; CT = conventional treatment scheme (ART), and MT = modified treatment scheme (VGC + ART) (Figure 2). All participants signed informed consent; samples from each group were recovered randomly for this study of patients that had complete follow-up visits with a frozen blood sample.

Once the patient entered the study protocol, blood samples were collected at the baseline (W_0_), week 4 (W_4_), and week 12 (W_12_). ART was started according to current Mexican Guidelines [28]. The CT group received ART since W_0_, and the MT group started VGC 900 mg twice a day starting at W_0_, and ART was initiated at W_4_, and they continued with both drugs across the follow-up (Figure 2). Chemotherapy (bleomycin and vincristine) was administrated to patients according to treating physician criteria.

In parallel, 10 MSM tested negative for HIV in the screening, and 10 MSM tested positive, asymptomatic with >350 CD4+ cells/mL; without KS and paired by age were included as a control (Figure 2). These participants assisted HIV screening at *Clinica Especializada Condesa*, the Mexico City specialized clinic for HIV diagnosis and care.

The median value of each evaluated parameter in the HIV-negative men is shown as a black dashed line in each graph. The value number is shown in supplementary data.

### 2.3. Cells

Peripheral blood mononuclear cells (PBMCs) were obtained from 8 mL of the blood by density gradient and cryopreserved until use, as previously reported [29]. Briefly, cryopreserve PBMCs were thawed and resuspended in phosphate-buffered saline (PBS) and counted, using a TC20™ Automated Cell Counter (Bio-Rad, Hercules, CA, USA). The trypan blue dye exclusion was used to determine PBMCs viability.

### 2.4. Multiparametric Flow Cytometry

PBMCs were prepared to evaluate cell surface marker expressions using monoclonal antibodies (mAbs) to CD3, CD56, CD16, CD57, CD27, CD279 (PD-1), and KLRG1. All the mAbs were provided by BioLegend (San Diego, CA, USA). The cells used for Fluorescence Minus One (FMO) condition were stained and acquired in parallel to identify background levels of staining; dead cells were omitted using viability staining Zombie Red Dye solution (BioLegend).

The data were acquired using a FACS Aria II flow cytometer (BD Biosciences, San Jose, CA, USA) equipped with the FACSDiva 6.1.3 software (BD Biosciences, San Jose, CA, USA). In each condition, at least 50,000 events were acquired per sample. The flow cytometry data file (FCS) was analyzed using Flow Jo (Flow Jo, LLC, Ashland, OR, USA) ™ v10.6.1. The frequency of NK and NKT cells was analyzed, and the mean value for fluorescence intensity (MFI) was calculated for all markers. A list of the antibodies used can be found in Table S1.

### 2.5. Enzyme-Linked Immunosorbent Assay (ELISA)

Plasma samples were stored at −70 °C until analysis. Soluble plasma levels of IL-15, PD-L1, PD-L2, and E-cadherin were quantified using a sandwich ELISA system and following the manufacturer’s recommendations (Table S1). All proteins were quantified by comparison with the corresponding standard curve, and the optical density was measured using a microplate reader spectrophotometer (Imark, Bio-Rad, Hercules, CA, USA) set to 450 nm.

### 2.6. Statistical Analysis

Data are shown as median with interquartile range (IQR, 25–75). Mann–Whitney U test was used to compare two groups, and the Kruskal–Wallis test with Dunnett’s post-test was used for multiple comparisons. *p* < 0.05 were considered statistically significant (GraphPad Software, Inc., San Diego, CA, USA).

## 3. Results

### 3.1. Baseline Clinic Characteristics of Study Populations

We enrolled 20 men with a disseminated KS diagnosis (KS/HIV patients) naïve to ART. They were followed up at 12 weeks and divided depending on the received scheme treatment, CT, and MT for this study. The median age for the CT group was 32 (26–45) years and 34 (27–41) years for MT group.

**Figure 3:**
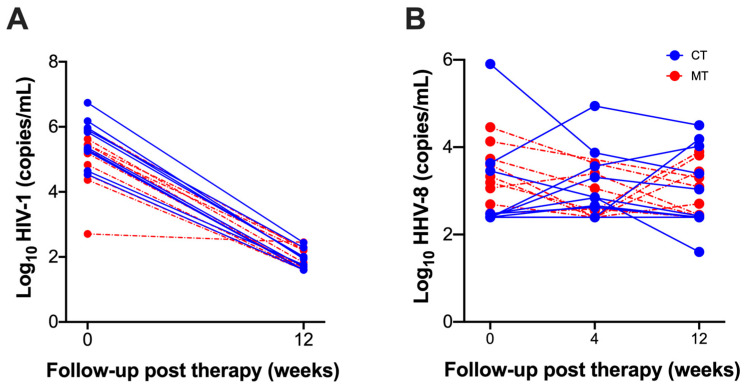
Viral loads of HHV-8 and HIV-1 in KS/HIV patients across follow-up. The viral load of (**A**) HIV-1 at the baseline (before ART, W_0_ for CT, and W_4_ for MT) and across the follow-up (weeks of ART, W_12_ for CT, and W_8_ for MT), and (**B**) HHV-8. Each point on the graph represents the viral load of each patient at a specific time of CT and MT groups.

Plasma viral load (VL) of HIV-1 and HHV-8 of KS/HIV patients was evaluated before ART and across the follow-up. HIV-1 viral load only is shown for the CT group at W_0_ and W_12_ (before and after 12 weeks of ART, respectively), and for the MT group at W_4_ and W_12_ (before and after 8 weeks of ART, respectively) in Table 2. HIV-1 VL reached almost undetectable levels in both CT and MT groups at W_12_; however, HHV-8 did not show a significant decrease even at W_12_. HHV-8 VL showed high variability across the follow-up in both groups (Figure 3A), whereas HIV-1 VL showed similar behavior, even though the MT group started ART at W_4_ (Figure 3B).

### 3.2. Plasma Levels of IL-15 Are Increased in KS/HIV Patients at the Baseline and Decreased across Follow-Up after Starting Treatment

**Figure 4:**
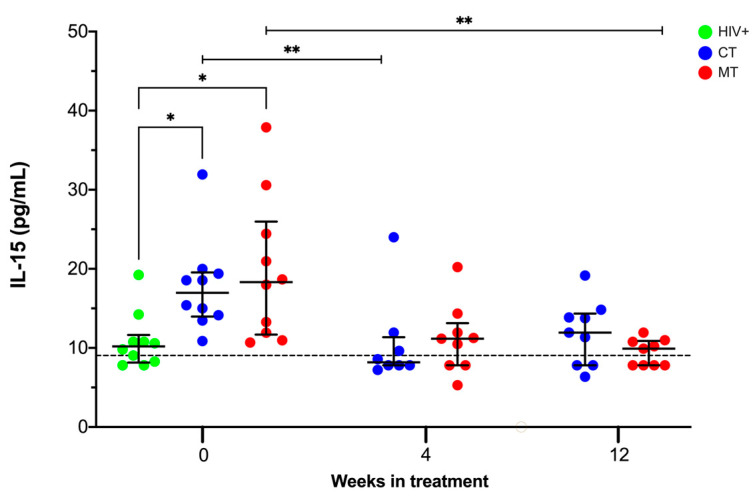
High circulating IL-15 levels at baseline in KS/HIV patients decreased after treatment. IL-15 levels were compared at the baseline (W_0_) between KS/HIV and HIV+ patients and across all follow-up weeks between each group. The black dashed line represents the median value of HIV-negative men. Green dots represent HIV+ asymptomatic men, blue dots represent CT and red MT participants. Statistical comparisons were performed using Kruskal–Wallis test, * *p* < 0.05, ** *p* < 0.01.

IL-15 is fundamental for the expansion and function of NK and NKT cells; thus, we evaluated the plasma levels of IL-15 in KS/HIV patients with CT and MT schemes. Both groups of KS/HIV patients increased the IL-15 level at W_0_ compared to HIV+ asymptomatic men [CT, *p* = 0.0318; MT, *p* = 0.0271]. However, across the clinical follow-up, KS/HIV patients with CT decreased IL-15 levels at W_4_ compared to W_0_ (*p* = 0.0020), whereas those KS/HIV patients that received MT decreased IL-15 level until the W_12_ compared to W_0_ (*p* = 0.0030) (Figure 4). These results show that KS/HIV patients increased IL-15 levels compared to HIV+, CT decreasing faster than MT. The median values and IQR of the IL-15 levels across follow-up and the multiple comparisons between groups are summarized in Table S2.

### 3.3. Frequency of NK Cells Is Affected by KSHV Infection, Even Though They Increased after Treatment with Either Scheme Treatment, While NKT Cells Frequency Only Increases in the MT Group

**Figure 5:**
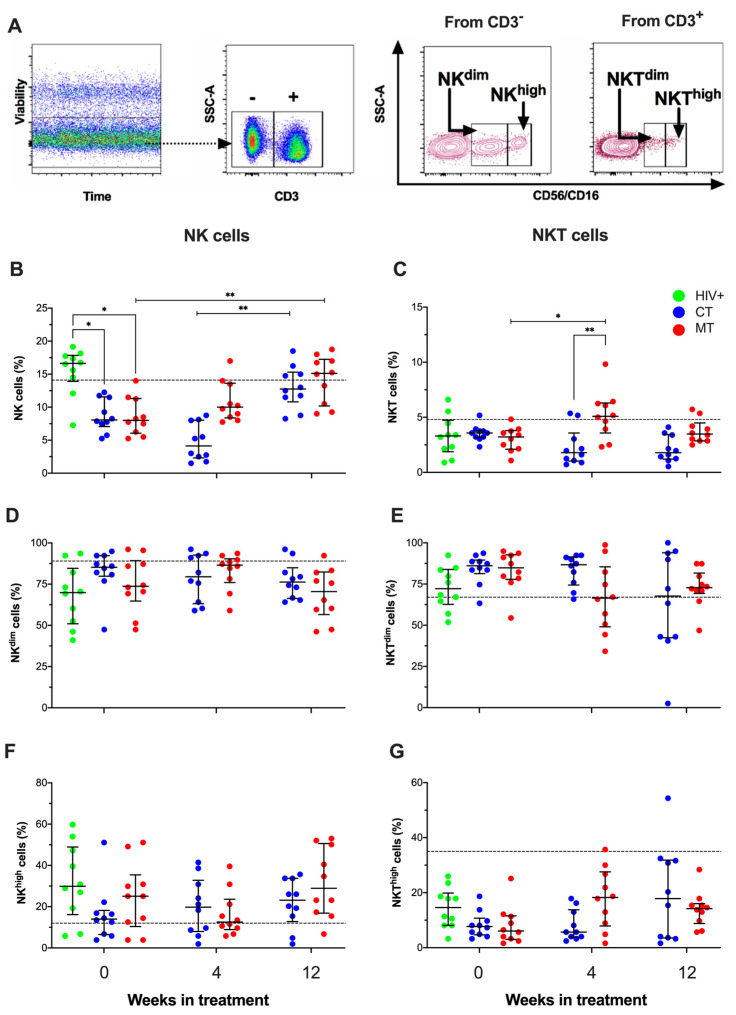
The frequency of NK cells decreased with the KSHV infection and increased after treatment with either scheme treatment. Singlets cells were identified through forward scatter (FSC-A versus FSC-H). Then, viable cells were selected, followed by forward scatter (FSC) and side scatter (SSC) dot plot to selected live lymphocytes (Dot plots not displayed). (**A**) From CD3-negatives or CD3-positives events, NK or NKT (dim/high) cells based on CD56/CD16 expression were identified. The frequency of NK (**B**) and NKT (**C**) cells and their subpopulations dim (**D**,**E**) and high (**F**,**G**) in HIV+ asymptomatic men and KS/HIV patients were analyzed as indicated. The black dashed line represents the median value of HIV-negative men. Green dots represent HIV+ asymptomatic men, blue dots represent CT and red MT participants. Statistical comparisons were performed using Kruskal–Wallis test, * *p* < 0.05, ** *p* < 0.01.

Based on the observed regulation of IL-15 during follow-up, we hypothesized that the frequency of NK and NKT cells is modified in a treatment scheme-dependent way. Therefore, the frequencies of total NK and NKT cells and subsets (CD56^dim^ and CD56^high^) were analyzed by flow cytometry; CD3- and CD3+ cells gates were delimited inside the gate of the viable cells. Then, the expression of CD56/CD16 was evaluated to identify the frequency of total NK (CD3-CD56/CD16+) and total NKT cells (CD3+CD56/CD16+); moreover, according to the intensity of CD56/CD16 expression, they were divided into dim and high subpopulations (Figure 5A).

Results indicated that KS/HIV patients had decreased the frequency of total NK cells at W_0_ compared to HIV+ asymptomatic men [16.9 (13.9–17.8); CT [8.1 (7.0–12.0), *p* = 0.093; MT 8.1 (6.2–11.1), *p* = 0.043], and both treatment schemes increased the frequency of total NK cells at W_12_ compared to W_0_ (Figure 5B). In addition, KS/HIV patients had a similar frequency to total NKT cells at W_0_ compared to HIV+ asymptomatic men; however, MT increased the frequency of total NKT cells at W_4_ compared to W_0_ ([5.3 (3.8–6.3) vs. 3.3 (2.3–3.7), *p* = 0.0197) and compared to CT at W_4_ ([5.3 (3.8–6.3) vs. 1.8 (1.1–3.6), *p* = 0.008)] (Figure 5C). Finally, we did not observe differences in the frequency of NK^dim^, NKT^dim^, NK^high^, and NKT^high^ subpopulations (Figure 5D–G, respectively). These data show that KS decreased the frequency of NK cells, and although the CT or MT scheme increased NK frequency at W_12_, only MT increased NKT cells at W_4_.

### 3.4. CD57 and CD27 Expression on NK Subsets Are Differentially Modified Depending on the Treatment Scheme in KS/HIV Patients

**Figure 6:**
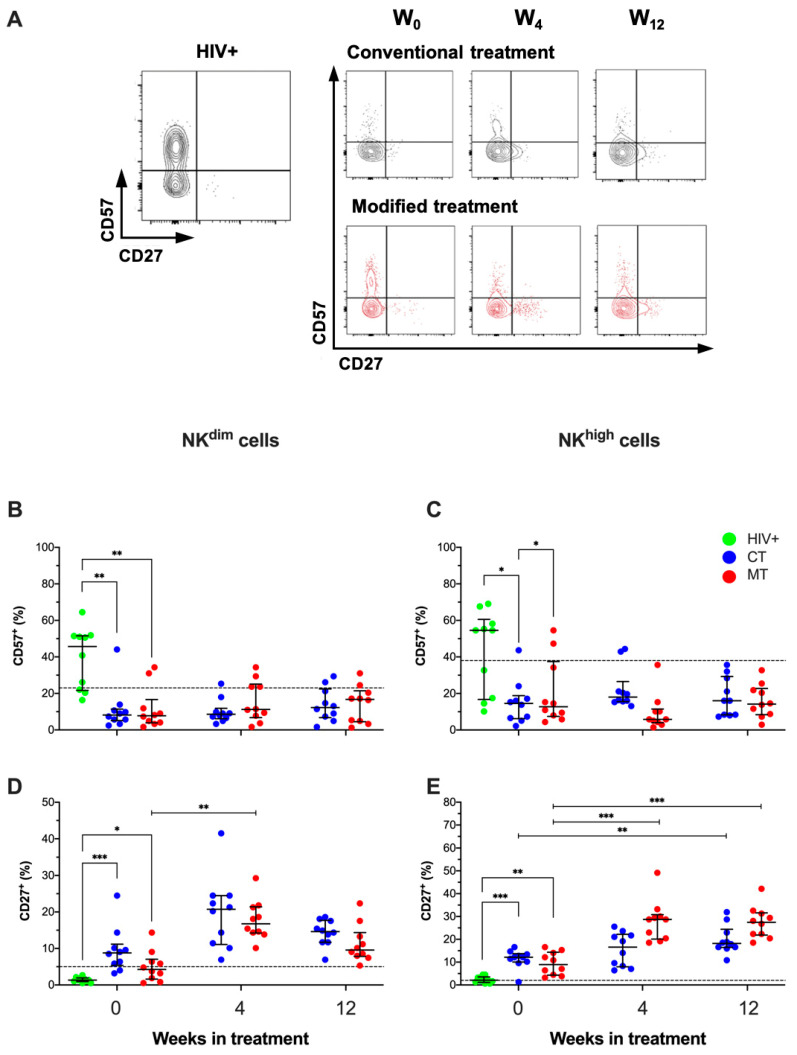
Differential expression of CD57 and CD27 on NK subsets depends on the treatment scheme in KS/HIV patients. (**A**) Representative density dot plots of NK cells expressing CD57 or CD27 in HIV+ and KS/HIV patients. Frequencies of CD57+ (**B**,**C**) and CD27+ (**D**,**E**) on both NK cells subsets were evaluated in HIV+ and KS/HIV patients across the follow-up weeks in each group. The black dashed line represents the median value of HIV-negative men. Green dots represent HIV+ asymptomatic men, blue dots represent CT and red MT participants. Statistical comparisons were performed using Kruskal–Wallis test, * *p* < 0.05, ** *p* < 0.01, *** *p* < 0.001.

Into the NK^dim^ and NK^high^ gates (Figure 5A), we evaluated the co-expression of CD57 and CD27 (Figure 6A). First, a comparison between HIV-negative and HIV+ asymptomatic patients was made. We observed that HIV+ patients showed a decline in the frequency of both NK^dim^ and NK^high^ positive to CD27 compared to HIV-negative men (Table S3).

Our data showed that KS/HIV patients at W_0_ had decreased the frequency of CD57+ NK^dim^ cells compared to HIV+ asymptomatic men [CT, *p* = 0.0034; MT, *p* = 0.0019] (Figure 6B). Similarly, the frequency of CD57+ NK^high^ cells was decreased in KS/HIV patients at W_0_ compared to HIV+ [CT, *p* = 0.0292; MT, *p* = 0.0422] (Figure 6C), and this profile is maintained across follow-up.

In contrast, the frequency of CD27+ NK^dim^ cells is higher in KS/HIV at W_0_ than HIV+ asymptomatic men [CT, *p* = 0.0001; MT, *p* = 0.0207]. Interestingly, MT induced a strong increase of CD27+ NK^dim^ cells at W_4_ compared to W_0_ [MT, *p* = 0.0001] (Figure 6D). Similarly, the frequency of CD27+ NK^high^ cells was increased in KS/HIV patients at W_0_ compared to HIV+ asymptomatic patients [CT, *p* = 0.0004; MT, *p* = 0.0043]. MT induced a sustained increase of CD27+ NK^high^ cells both at W_4_ and W_12_ compared to W_0_ [W_4_, *p* = 0.0004; W_12_, *p* = 0.0005], whereas CT only increased the frequency of CD27+ NK^high^ cells at W_12_ compared to W_0_ (*p* = 0.0096) (Figure 5E). The results shows that CD57+ NK^dim^ and CD57+ NK^high^ are regulated contrary to CD27+ NK^dim^ and CD27+ NK^high^, and it was favored mainly by the MT scheme since W_4_. The median values and IQR of the frequencies of CD57+ and CD27+ on NK cells subpopulations across follow-up are summarized in Table S4.

### 3.5. PD-1 and KLRG1 Expression on the NK Cells Subsets Are Differentially Modified Depending on the Treatment Scheme in KS/HIV Patients

PD-1 expression can be induced by HIV-1 and HHV-8 on NK cells, and reports indicated that KLRG1 expression on cytotoxic cells enhances viral control. Thus, we examined the MFI of PD-1 and KLRG1 on NK^dim^ (CD27+ and CD57+) and NK^high^ (CD27+ and CD57+) cell subpopulations by flow cytometry.

**Figure 7:**
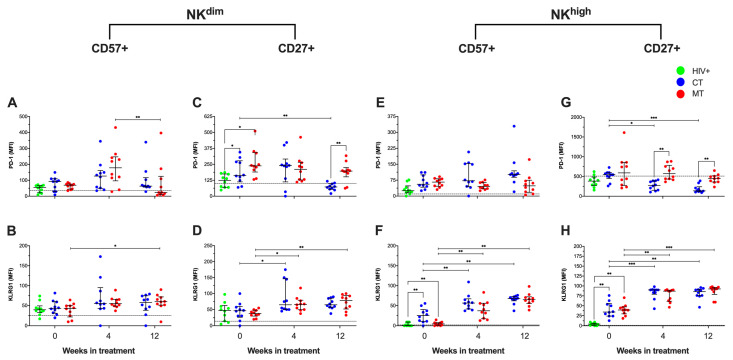
Expressions of the immune checkpoint receptors PD-1 and KLRG1 are differentially modified depending on the treatment scheme in KS/HIV patients. PD-1 and KLRG1 expression (MFI) on CD57+ and CD27+ cells of NK^dim^ (**A**,**B**,**E**,**F**) and NK^high^ (**C**,**D**,**G**,**H**) cell subsets of HIV+ and KS/HIV patients were evaluated across the follow-up weeks in each group. The black dashed line represents the median value of HIV-negative men. Green dots represent HIV+ asymptomatic men, blue dots represent CT and red MT participants. Data are represented as median values and IQR. Statistical comparisons were performed using Kruskal–Wallis test, * *p* < 0.05, ** *p* < 0.01, *** *p* < 0.001.

KS/HIV patients, using the MT scheme, decreased the MFI of PD-1 on CD57+ NK^dim^ cells from W_4_ to W_12_ (*p* = 0.0357) (Figure 7A), whereas the MFI of KLRG1 increased at W_12_ compared to W_0_ (*p* = 0.0221) (Figure 7B). KS/HIV at W_0_ had higher MFI of PD-1 on CD27+ NK^dim^ cells than HIV+ patients [CT, *p* = 0.01; MT, *p* = 0.0117], but CT scheme decreased the PD-1 expression at W_12_ compared to W_0_ (*p* = 0.0038). In contrast, the MT scheme maintains the PD-1 expression higher than CT scheme (*p* = 0.0015) (Figure 7C). On CD27+ NK^dim^ cells, both CT and MT increased the MFI of KLRG1 at W_4_ compared to W_0_ (*p* = 0.02 and *p* = 0.0100, respectively), but only MT maintained high KLRG1 expression at W_12_ compared to W_0_ (*p* = 0.0027) (Figure 7D).

Regarding NK^high^ cell subpopulations, our data showed that CD57+ NK^high^ cells from KS/HIV at W_0_ had higher MFI of PD-1 than HIV+ asymptomatic men [CT, *p* = 0.0118; MT, *p* = 0.0085]. All treatments schemes modified this expression during the follow-up (Figure 7E). Similarly, MFI of KLRG1 was increased on CD57+ NK^high^ cells from KS/HIV at W_0_ compared to HIV+ asymptomatic men [CT, *p* = 0.0001; MT, *p* = 0.0411], and both treatment schemes increased the MFI of KLRG1 at W_4_ (CT, *p* = 0.0162 and MT, *p* = 0.0133) and W_12_ (CT, *p* = 0.0002 and MT, *p* = 0.0001) compared to W_0_ (Figure 7F). On the CD27+ NK^high^ cells, we observed that CT affected the MFI of PD-1 both at W_4_ and W_12_ compared to W_0_ (*p* = 0.0144 and *p* = 0.0002, respectively), whereas the MT scheme maintained a higher level of PD-1 expression than CT both at W_4_ (*p* = 0.0049) and W_12_ (*p* = 0.0070) (Figure 7G). MFI of KLRG1 was increased on CD27+ NK^high^ cells from KS/HIV at W_0_ compared to HIV+ asymptomatic men [CT, *p* = 0.0004; MT, *p* = 0.0004], and both treatment schemes induced a stronger increase of KLRG1 at W_4_ (CT, *p* = 0.0007 and MT, *p* = 0.0029) and W_12_ (CT, *p* = 0.0055 and MT, *p* = 0.002) compared to W_0_ (Figure 7H). The median values and IQR of the MFI of PD-1 and KLRG1 on NK cells subpopulations across follow-up and the multiple comparisons between groups are summarized in Tables S5 and S6.

### 3.6. MT Scheme Decreased CD57+ NKT^high^ Cells, Whereas Increased the Frequency of CD27+ NKT^dim^ in KS/HIV Patients

**Figure 8:**
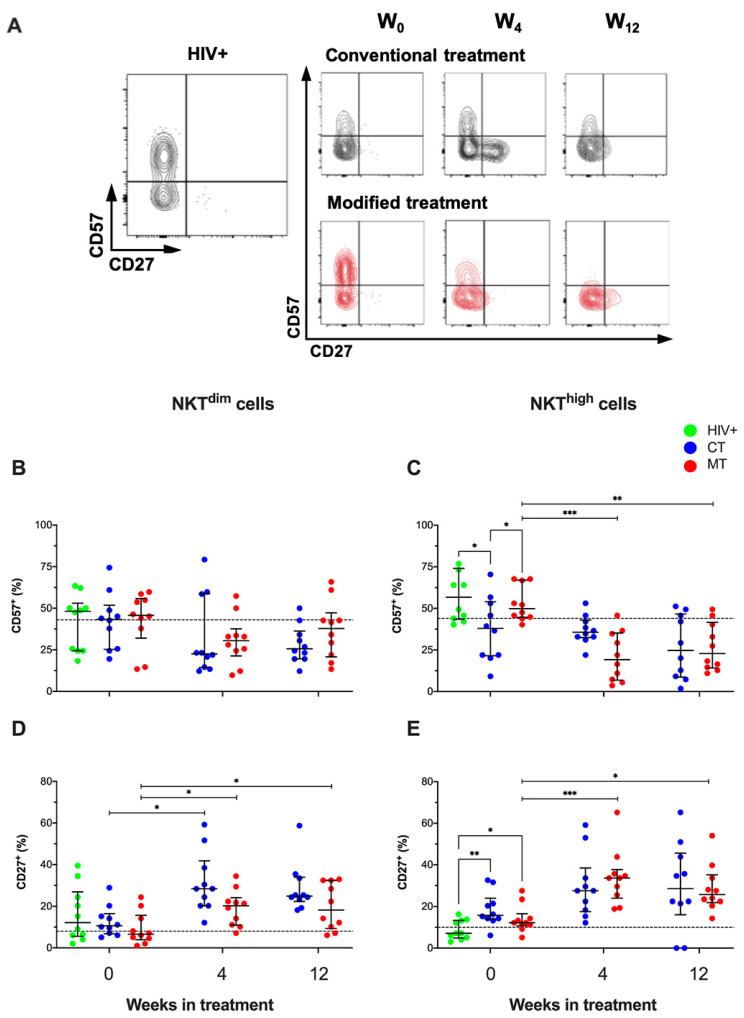
MT scheme decreased CD57+ NKT^high^ cells and increased CD27+ NKTdim frequency in KS/HIV patients. (**A**) Representative density dot plots of NKT cells expressing CD57 or CD27 in HIV-negative men, HIV+, and KS/HIV patients. Frequencies of CD57+ (**B**,**C**) and CD27+ (**D**,**E**) on both NK cells subsets were evaluated across the follow-up in each group. The black dashed line represents the median value of HIV-negative men. Green dots represent HIV+ asymptomatic men, blue dots represent CT and red MT participants. Data are represented as median values and IQR. Statistical comparisons were performed using the Kruskal–Wallis test, * *p* < 0.05, ** *p* < 0.01, *** *p* < 0.001.

In NKT^dim^ and NKT^high^ gates, we evaluated the co-expression of CD57+ and CD27+ (Figure 8A). First, a comparison between HIV-negative men and HIV+ patients was made, and we observed that HIV+ patients and HIV-negative men showed a similar frequency of both NKT^dim^ and NKT^high^ positive to CD27 and CD57 (Table S7).

Our data showed that the frequency of CD27+ NKT^dim^ in KS/HIV patients was similar across the follow-up weeks in each group (Figure 8B). However, KS/HIV patients at W_0_ decreased the frequency of CD57+ NKT^high^ cells compared to HIV+ asymptomatic men [CT, *p* = 0.0116; MT, *p* = 0.0283], and MT decreased the frequency of CD57+ NKT^high^ cells at W_4_ (*p* = 0.0003) and W_12_ compared to W_0_ (*p* = 0.0049) (Figure 8C). Regarding the frequency of CD27+ NKT^dim^ cells, CT increased the frequency of this subpopulation at W_4_ compared to W_0_ (*p* = 0.0287); similarly, MT increased the frequency of CD27+ NKT^dim^ at W_4_, although this profile was maintained at W_12_ compared to W_0_ [W_4_ vs. W_0_, *p* = 0.0397; W_12_ vs. W_0,_ *p* = 0.0357] (Figure 8D). Finally, KS/HIV patients had higher frequency of CD27+ NKT^high^ cells than HIV+ asymptomatic men [CT, *p* = 0.0034; MT, *p* = 0.01]. MT induced a sustained increase of CD27+ NKT^high^ cells at W_4_ and W_12_ compared to W_0_ [W_4_ vs. W_0_, *p* = 0.0008; W_12_ vs. W_0_, *p* = 0.0212] (Figure 8E). Our results show that CD57+ NKT^high^ was regulated contrary to CD27+ NKT^dim^ and CD57+ NKT^high^, and it was favored mainly by the MT scheme since W_4_. The median values and IQR of the frequencies of CD57+ and CD27+ on NKT cells subpopulations across follow-up and the multiple comparisons between groups are summarized in Table S8.

### 3.7. PD-1 Expression Is Decreased, While KLRG1 Expression Is Increased on CD27+ NKT^dim^ Cells with MT

Chronic infections can increase PD-1 expression on NKT cells [30]. Thus, we examined PD-1 and KLRG1 expression on NKT^dim^ (CD27+ and CD57+) and NKT^high^ (CD27+ and CD57+) cells subpopulations by flow cytometry.

**Figure 9:**
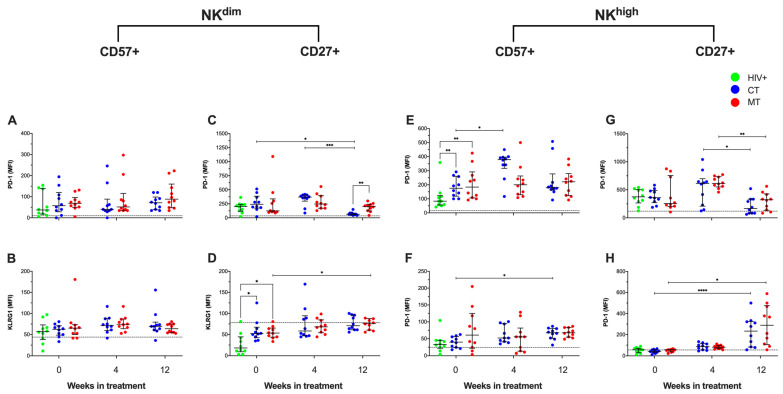
PD-1 expression was affected, while KLRG1 expression increased on CD27+ NKT^dim^ cells**.** PD-1 and KLRG1 expression (MFI) on CD57+ and CD27+ cells of NKT^dim^ (**A**,**B**,**E**,**F**) and NKT^high^ (**C**,**D**,**G**,**H**) cell subsets were evaluated across the follow-up weeks in each group. The black dashed line represents the median value of HIV-negative men, green dots represent HIV+ asymptomatic men. Green dots represent HIV+ asymptomatic men; blue dots represent CT and red MT participants. Data are represented as median values and IQR. Statistical comparisons were performed using Kruskal–Wallis test, * *p* < 0.05, ** *p* < 0.01, *** *p* < 0.001, **** *p* < 0.0001.

We observed that the MFI of PD-1 and KLRG1 on CD57+ NKT^dim^ cells from KS/HIV patients was similar to HIV+ asymptomatic men, and both treatment schemes did not modify the expression of PD-1 and KLRG1 across the follow-up weeks (Figure 9A,B, respectively). On CD27+ NKT^dim^ cells, PD-1 expression was lower in KS/HIV patients using CT at W_12_ compared to either W_0_ or W_4_ (*p* = 0.0230 and *p* = 0.0001, respectively) (Figure 9C). Whereas, MFI of KLRG1 was higher in KS/HIV than HIV+ asymptomatic men [CT, *p =* 0.0407; MT, *p* = 0.0405], and it increased at W_12_ in KS/HIV patients with MT scheme, compared to W_0_ (*p* = 0.0196) (Figure 9D).

Regarding CD57+ NKT^high^ cells, the MFI of PD-1 was higher in KS/HIV than in HIV+ asymptomatic men [CT, *p =* 0.0021; MT, *p* = 0.0010]; and CT increased the PD-1 expression on CD57+ NKT^high^ cells at W_4_ compared to W_0_ (*p* = 0.0287) (Figure 9E). Similarly, the CT scheme increased the MFI of KLRG1 at W_12_ compared to W_0_ (*p* = 0.0175) (Figure 9F). Both treatments affected the PD-1 expression on CD27+ NKT^high^ cells at W_12_ compared to W_4_ (CT, *p* = 0.0133; MT, *p* = 0.0069) (Figure 9G). On the contrary, MT increased the KLRG1 expression on CD27+ NK^high^ cells at W_12_ compared both to W_0_ and W_4_ (*p* = 0.0001 and *p* = 0.0229, respectively); moreover, CT also induced increased expression of KLRG1 but only W_12_ compared to W_0_ (*p* = 0.0001) (Figure 9H). The median values of the MFI of PD-1 and KLRG1 on NKT cells subpopulations across follow-up and the multiple comparisons between groups are summarized in Tables S9 and S10.

### 3.8. PDL-1 Plasma Levels Are Increased in KS/HIV Patients, While PDL-2 and E-Cadherin Plasma Levels Are Not Modified

**Figure 10:**
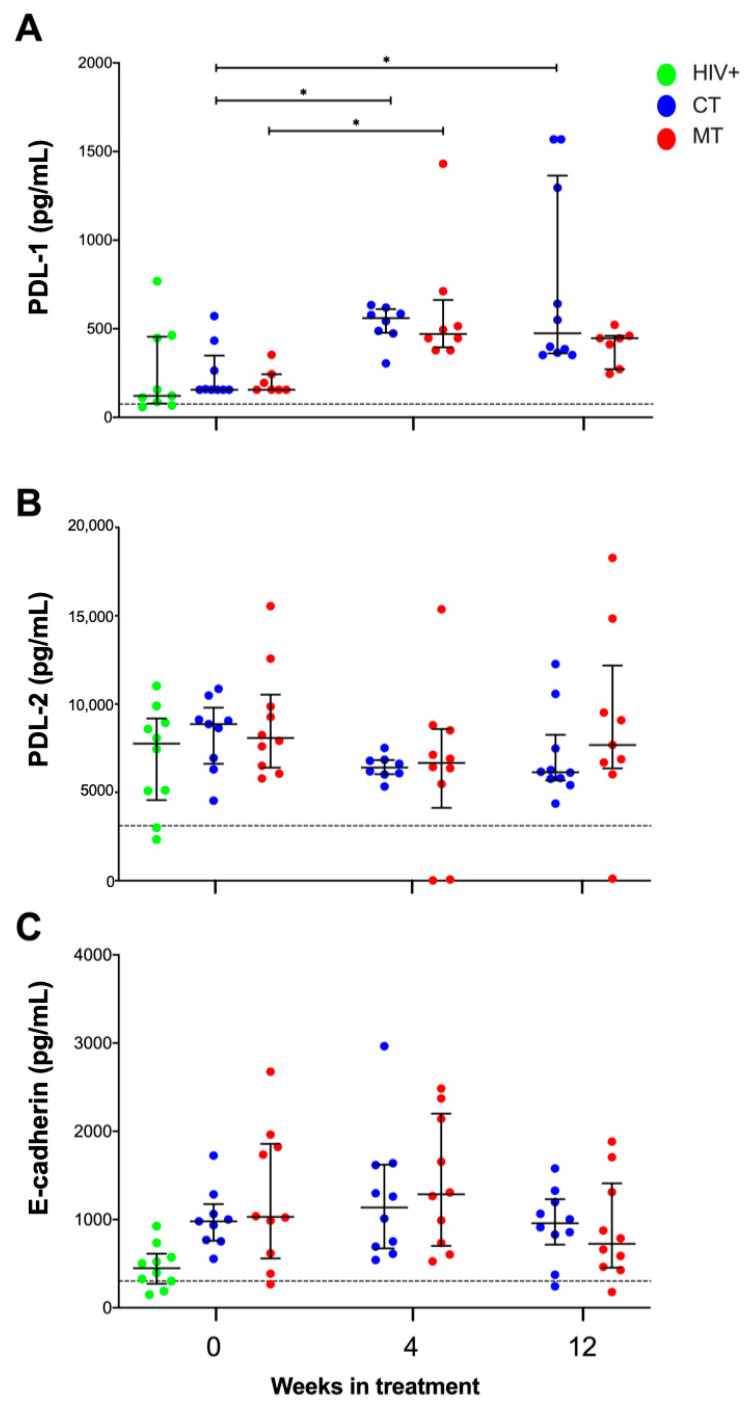
Plasma levels of PDL-1 increased, while PDL-2 and E-cadherin plasma levels were not modified in KS/HIV patients. PD-L1, PD-L2 (**A**,**B**), and E-cadherin (**C**) were measured in plasma at the baseline (W_0_) between KS/HIV and HIV+ patients and across the follow-up in each group. The black dashed line represents the median value of HIV-negative men. Green dots represent HIV+ asymptomatic men; blue dots represent CT and red MT participants. Data are represented as median values and IQR. Statistical comparisons were performed using the Kruskal–Wallis test, * *p* < 0.05.

It is known that the PD-1/PDL-1 or PDL-2 axis plays an essential role in immunity against viral infections, whereas E-cadherin/KLRG1 axis regulates the activation of NK and NKT cells in viral infections [31,32]. Therefore, PDL-1, PDL-2, and E-cadherin plasma levels from KS/HIV patients were measured across the follow-up. Our data showed that PDL-1, PDL-2, and E-cadherin levels increased in KS/HIV patients compared with HIV-negative men (Table S11). However, they were not different compared to the HIV+ asymptomatic men group (Figure 10A–C, respectively).

The analysis of plasma levels across the follow-up showed that KS/HIV patients with CT increased the PDL-1 level at W_4_ [560.2 pg/mL (477.3–611.0), *p* = 0.0145)] and W_12_ [474.3 pg/mL (362.0–1364.0), *p* = 0.0330] compared to baseline [157.3 pg/mL (138.0–468.0)]. In contrast, MT only increased PDL-1 plasma levels at W_4_ compared with baseline [446.5 pg/mL (379.0–515.1) vs. 156.0 (156.0–244.0), *p* = 0.0189] (Figure 10A). We did not observe differences of PDL-2 and E-cadherin soluble levels (Figure 10B,C, respectively).

## 4. Discussion

In this study, we investigated the frequency and phenotype of NK and NKT cells in KS/HIV patients across the first 12 weeks of VGC treatment (modified treatment scheme); moreover, it was compared to patients that received a conventional treatment scheme.

Our data provide first-time evidence that VGC as an add-on therapy in KS/HIV induces changes in the profile of immune cells within 12 weeks of treatment. Patients showed heterogeneity in HHV-8 VL at baseline; noteworthy, VGC influenced the HHV8 VL, there was a decrease at W4, and 30% of patients had an increase of HHV-8 VL at W_12_. In contrast, the CT group patients who had a low VL at W_0_ controlled the viremia until W_12_, but among them, 40% of patients increased the HHV-8 VL from W_4_ until W_12_ (Figure 3A). Regarding HIV VL, almost all patients enrolled decreased; nevertheless, the MT group reached undetectable levels (40 copies/mL) at 8 weeks of ART (it corresponds to 12 weeks of follow-up), while the CT group reached this level at 12 weeks of ART (Figure 3B). These results suggest that VGC could also help control the HIV VL, even though the MT group started ART at W_4_. Thus, additional studies are necessary to evaluate the effect of MT treatment at a long time of treatment (at least 24 weeks) to confirm if this scheme has a positive effect on the HHV-8 VL in KS/HIV patients. In fact, VGC as a complementary therapy in KS/HIV controls the viral replication of HHV-8 [33]. Therefore, we consider it necessary to understand host immune responses during VGC on cell subpopulations that play a fundamental antiviral role as NK and NKT and not solely the effect on HHV-8 VL. Thus, VGC may modulate other immune response mechanisms as the inflammatory process through regulation in the frequency of cell subpopulations. In this regard, reports showed that HIV patients that used a treatment scheme of oral plus implant ganciclovir reduce the incidence of cytomegalovirus disease and delay the progression of the inflammatory process as retinitis [34].

Reports indicate that antiretroviral therapy for HIV infection impacts the frequency and function of cytotoxic cells. Recently, it has been reported that the early initiation of ART in children with perinatally acquired HIV preserves the NK cells compartment and is associated with a lower HIV reservoir. In contrast, in vitro, it has been reported that histone deacetylase inhibitors up-modulate the expression of ligands such as NKG2D on NK cells. Thus, it is necessary for NKG2D-mediated viral suppression by NK cells [35,36].

We observe that the coinfection HIV/HHV-8 increases IL-15 levels (at the baseline); however, it gradually decreases in the MT group (VGC plus ART). It is well established that IL-15 is necessary to maintain and activate NK and NKT cells subpopulations; in HIV patients, the viral infection causes activation and expansion of NK cells, and the NK cells subpopulations show an abnormal frequency [37,38]. Moreover, IL-15 levels are increased and positively correlated to high HIV VL [39].

In contrast to what was expected, KS/HIV patients had a lower NK cell frequency than HIV+ asymptomatic individuals. Although the frequency of NKT cells was not modified at baseline, it increased at W_4_ with MT. In agreement with other studies, we confirmed that the proportions of CD56^dim^ and CD56^high^ NK cell subpopulations are not different in KS/HIV patients [40]. Regarding IL-15 levels, KS/HIV patients have higher levels than HIV+ asymptomatic patients, suggesting that HHV-8 promotes IL-15 delivering. We suggest that VGC in KS/HIV patients is helpful to regulate the IL-15 level similarly to ART at baseline, and it is beneficial to maintain the normal frequency of NK cells at W_12_. These results highlight the need to evaluate the IL-15 effect on the functionality of these subpopulations in KS/HIV patients.

Moreover, patients have extreme elevations of HHV-8 viral load and IL-6 level; it has been reported that in HIV/HHV-8 coinfection, IL-4, IL-6, and IL-10 levels are increased [41,42]. Noteworthy, KS/HIV patients with the MT scheme had a better clinical evolution than those who received only ART. This study did not evaluate if the MT scheme favors a cytokine profile. However, because the cytokine profile plays a central role in activating diverse immune cells subpopulations and promotes viral replication, inducing the lytic phase of HHV-8 through KSHV/Rta transcriptional activator, a Rta gene homolog encoded by Epstein–Barr virus in lymphoid cells, it should be considered in a future study if IL-15 promotes viral replication of HHV-8 [43].

Previous studies have reported that the chronic inflammatory process might drive the expansion of CD57+ and CD27+ NK cells, and HIV+ patients increased these subpopulations CD57+ after a long time on ART. However, it has not yet been clarified if the therapy based on VGC modifies CD57+ and CD27+ NK cells [44,45,46]. We found that KS/HIV patients decreased CD57+ NK cells numbers compared to HIV+ patients naïve to ART, and CD57+ NK cells frequency at W_4_ and W_12_ in both treatment schemes is similar to W_0_. Based on these data, we hypothesized that the coinfection with HHV-8 affects CD57+ NK cells restoration.

Regarding NKT cells, HIV infection causes a significant reduction in the NKT circulating number [47]. We found that compared to HIV+, KS/HIV patients have a low frequency of CD57+ NK^dim^, CD57+ NK^high^, and CD57+ NKT^high^, suggesting that the HHV-8 infection affects CD57 expression on NK and NKT cells subpopulations. The MT scheme did modify the frequency of CD57+ NK and CD57+ NKT, and this regime only increased the frequency of NK and NKT cells subpopulations of CD27+. Probably, the VGC treatment is helpful for early immune restoration, where CD27 is necessary to regulate cytotoxic functions in these subpopulations.

KS/HIV patients treated with VGC modified the frequency of NK and NKT cells subpopulations, suggesting that HIV/HHV-8 coinfection induces a dysfunction on NK and NKT cells, which could be reverted during the first 3 months of treatment. Our data showed a differential expression of molecules involved in the activation and development of NK and NKT cells when VGC is used as an add-on therapy according to HHV-8 VL decrease. The NK and NKT cell restoration could probably be associated with add-on therapy with VGC.

It has been suggested that the responsiveness of NK cells is related to PD-1 expression [26]. Here we found that PD-1 expression on NK and NKT cell subpopulations was modified during the follow-up of the patients, and changes were independent of the treatment regimen used; it is probably associated with a chronic activation caused by HIV. Furthermore, it has been reported that KLRG1 expression on cytotoxic cells is related to a rapid viral clearance [48]. We observed that KLRG1 expression is increased mainly at W_12_ using MT, suggesting that MT is helpful to control HHV-8 VL better. Reports indicate that KLRG1+ NK cells regulate their cytotoxicity function through the KLRG1/E-cadherin axis; additionally, HIV+ patients have increased levels of E-cadherin even after ART [49,50]. Although we did not observe differences in the E-cadherin level between patient groups, KS/HIV patients had a two-fold higher median value of E-cadherin level than HIV+ asymptomatic patients (1029.7 vs. 447.9, respectively) at baseline, and it was maintained at W_4_.

It has been documented that PD-L1, PD-L2, and E-cadherin can up-regulate genes in exhausted NK cells, impairing their function through PD-1 and KLRG1 receptors [51,52]. We found that only PD-L1 increased on KS/HIV patients at W_4_ in both treatment regimes, highlighting that the PD-L1 level was sustained until W_12_ in CT, while we did not observe differences with MT. Thus, this immune microenvironment activates immune system cells, including NK and NKT cells; together, our data suggest that NK cells have a reduced ability to induce a response against HHV-8 in HIV coinfected patients. Thus, our data showed that the use of VGC and ART in KS/HIV patients induces changes in the NK and NKT cells phenotype in the first weeks of treatment. These changes suggest that it could be considered as an alternative scheme of treatment in these patients, which usually show heterogeneous clinical characteristics due to the presence of OIs.

As was discussed by Coen N et al. [53]; the information to establish standard therapeutic guidelines for the management of KSHV-associated diseases is limited. The treatment for KS patients depends on several parameters, such as the tumor location and variant of KS, rate of progression, distribution of the lesions, severity of the symptoms, and immune competence. Consequently, efforts should be addressed in the search for therapeutic alternatives in the use of effective and selective antivirals; in this regard, we postulate that VGC is also helpful to modulate the immune response, which induces a better outcome in KS/HIV patients. Furthermore, it is well established that high HHV-8 VL levels are associated with disease severity and mortality [54]; HHV-8 replication correlates with extensive KS and disease progression [55]; therefore, VGC therapy could be relevant to counteract the KS progression and also help in patients who could have a relapse. Furthermore, other tumors and diseases caused by the HHV-8, such as the form of multicentric Castleman disease (MCD) and KSHV inflammatory cytokine syndrome, could be counteracted by limiting HHV-8 replication with the VGC. Even more important, VGC could significantly reduce the episodes of IRIS-KS and, therefore, the attributable mortality in the KS/HIV patients.

## 5. Conclusions

Clinical evidence showed that VGC-like add-on therapy for HHV-8 infection is helpful in disseminated KS. Nevertheless, it remains unclear if KS/HIV patients treated with VGC plus ART have good clinical evolution, where immune cells such as NK and NKT cells could be playing an important role. This study shows that VGC treatment, which specifically regulates the CD57 and CD27 expression on NK and NKT cells, could counteract HIV/HHV-8 coinfection resistance. However, further studies are needed to elucidate the contribution of NK and NKT cells in immune restoration on KS/HIV patients and the relationship between VGC treatment and the functional ability of NK and NKT cells during the follow-up period.

## Figures and Tables

**Table 1:** Characteristics of KS/HIV patients.

Parameters	All, *n* = 20	CT, *n* = 10	MT, *n* = 10
Age at enrollment median (IQR)	33 (26–41)	32 (26–45)	34 (27–41)
Smoker status			
No Current		8 (80%) 2 (20%)	6 (60%) 4 (40%)
KS severity		disseminated	disseminated
Concomitan coinfections			
	Syphilis	1 (10%)	2 (20%)
	Neurosyphilis	0	2 (20%)
	Histoplasmosis	1 (10%)	1 (10%)
	*Mycobacterium avium complex*	1 (10%)	2 (20%)
	Parasites	2 (20%)	1 (10%)
	*Helicobacter pylori*	2 (20%)	3 (30%)
EBV viremia			
W_0_		9 (90%)	10 (100%)
W_12_		8 (80%)	10 (100%)
CMV viremia			
W_0_		8 (80%)	6 (60%)
W_12_		1 (10%)	0
CD4 count cells/mL, median (IQR)			
W_0_	69.5 (33.5–192.8)	80.50 (36.5–212.0)	64.0 (27.8–195.8)
W_12_	103.5 (51.8–217.3)	156.5 (39.8–253.3)	64.5 (57.3–199.5)
CD8 count cells/mL, median (IQR)			
W_0_	972.5 (350.3–1223.0)	808.5 (335.8–1062.0)	1167.0 (386.3–1898.0)
W_12_	554.0 (324.5–718.0)	536.0 (344.8–637.8)	582.5 (236.5–1017.0)

CT = conventional treatment (ART), MT = modified treatment (VGC + ART). EBV, Epstein–Barr Virus; CMV, cytomegalovirus. Data are shown as percentages for EBV and CMV viremia in the CT or MT group.

**Table 2:** Viral loads of HIV-1 and HHV-8 in KS/HIV patients.

Tx	HIV-1 (Copies/mL)			HHV-8 (Copies/mL)			
	W_0_	W_12_	*p*	W_0_	W_4_	W_12_	*p*
CT	457,489 (144,567–1,067,196)	72 (40–124)	****	272 (218–3217)	256 (75–504)	675 (40–11,740)	ns
MT	W_4_ 176,115 (30,619–318,529)	W_12_ 56 (40–176)	****	1846 (445–7446)	2941 (57–12,923)	1232 (272–4620)	ns

CT = conventional treatment (ART), MT = modified treatment (VGC + ART). HIV-1, Human immunodeficiency virus 1; HHV-8: Human herpesvirus-8. Data are represented as median with interquartile range (IQR, 25–75). Statistical comparisons were performed using Kruskal–Wallis test to compare W_0_ with W_12_; **** *p* < 0.0001; ns, not significant.

## Data Availability

The data presented in this study are available in the article.

## Supplementary Materials

The following supporting information can be downloaded at: https://www.mdpi.com/article/10.3390/cancers14020412/s1, Table S1. Antibodies used for flow cytometry and ELISA; Table S2. IL-15 levels in KS/HIV patients across follow-up; Table S3. Frequency of NK cells subsets in control groups included in this study; Table S4. Frequencies of CD57+ and CD27+ on NK cells subpopulations across follow-up in KS/HIV patients; Table S5. PD-1 on the surface of circulating NK cells subsets; Table S6. KLRG1 on the surface of circulating NK cells subsets; Table S7. Frequency of NKT cells subsets in control groups included in this study; Table S8. Frequencies of CD57+ and CD27+ on NKT cells subpopulations across follow-up in KS/HIV patients; Table S9. PD-1 on the surface of circulating NKT cells subsets; Table S10. KLRG1 on the surface of circulating NKT cells subsets and Table S11. Plasma levels of PD-L1, PD-L2, E-cadherin, and IL-15.
