# Supplementary material for: Valganciclovir as Add-On Therapy Modifies the Frequency of NK and NKT Cell Subpopulations in Disseminated Kaposi Sarcoma Patients
Source: Cancers (Basel). 2022 Jan 14;14(2):412. doi: 10.3390/cancers14020412 (PMC8773484; doi:10.3390/cancers14020412)
Supplement: Supplementary file 1 [file cancers-14-00412-s001.zip › cancers-1523616-supplementary.pdf]

cancers

MDPI

Article

Valganciclovir as Add-On Therapy Modifies the Frequency of NK and NKT Cell Subpopulations in Disseminated Kaposi Sarcoma Patients

Julio Flores-Gonzalez [1,†], Lucero A. Ramon-Luing [1,†], Ranferi Ocaña-Guzman [1], Ivette Buendia-Roldan [2], Beda Islas-Muñoz [3], Patricia Volkow-Fernández [3] and Leslie Chavez-Galan [1,*]

1 Laboratory of Integrative Immunology, Instituto Nacional de Enfermedades Respiratorias Ismael Cosio Villegas, Mexico City 14080, Mexico; jfloresg1707@alumno.ipn.mx (J.F.-G.); ramonluing@yahoo.com.mx (L.A.R.-L.); arocana@iner.gob.mx (R.O.-G.)
2 Laboratory of Translational Research in Aging and Pulmonary Fibrosis, Instituto Nacional de Enfermedades Respiratorias Ismael Cosio Villegas, Mexico City 14080, Mexico; ivettebu@yahoo.com.mx
3 Infectious Diseases Department, Instituto Nacional de Cancerología, Mexico City 14080, Mexico; bedaislas@gmail.com (B.I.-M.); pvolkowf@gmail.com (P.V.-F.)
* Correspondence: lchavez_galan@iner.gob.mx or lchavezgalan@gmail.com; Tel. +52-555-487-1700 (ext. 5270)
† These authors contributed equally to this work.

Supplementary Material

Table S1. Antibodies used for flow cytometry and ELISA.

Antibody	Conjugate to	Clone/Catalog [1]	Company
CD3	APC-Cy7	OKT3	Biolegend
CD56	PE	B159	BD Pharmingen
CD16	PE	3G8	BD Pharmingen
CD57	Pe-Cy7	HNK-1	BD Pharmingen
CD27	PerCP-Cy5.5	O323	BD Pharmingen
KLRG1	FITC	2FI/KLRG1	Biolegend
CD279	APC	EH12.2H7	Biolegend
CD25	BV421	BC96	Biolegend
Viability	PE TexRed	NA‡	Biolegend
IL-15	NA‡	435104	Biolegend
sPD-L1	NA‡	DY156	R&D System
sPD-L2	NA‡	DY1224	R&D System
E-cadherin	NA‡	DY648	R&D System

[1] The clone is indicated for flow cytometry antibodies and the catalog number for ELISA. NA, not applicable.

Table S2. IL-15 levels in KS/HIV patients across follow-up.

	IL-15 (pg/mL)		
	**W_0**	**W_4**	**W_{12}**
**CT**	18.60 (13.8-19.7)	8.6 (7.8-22.8)	12.8 (7.8-15.9)
**MT**	18.3 (11.7-26.0)	11.2 (7.8-15.8)	10.06 (7.8-11.21)

Data are represented as median with interquartile range. CT=conventional treatment (ART), MT= modified treatment (VGC+ART).

Table S3. Frequency of NK cells subsets in control groups included in this study.

Subsets	HIV-Negative Men	HIV+ Asymptomatic Men	*p*
NK dim	88.9 (86.8 to 90.0)	70.3 (51.5 to 84.0)	ns
CD57+	24.3 (19.3 to 33.3)	45.9 (22.7 to 52.0)	ns
CD27+	4.7 (3.8 to 5.1)	1.7 (1.4 to 2.3)	0.0007
NK high	11.1 (9.9 to 13.2)	29.7 (15.9 to 48.5)	ns
CD57+	37.6 (22.1 to 44.8)	54.3 (17.4 to 59.7)	ns
CD27+	4.9 (4.6 to 8.2)	1.9 (1.5 to 4.8)	0.0047

Data are represented as median with interquartile range. Statistical comparisons were performed using Mann–Whitney U test, CI 95%. (ns: not-significant). HIV, Human immunodeficiency virus 1.

Table S4. Frequencies of CD57+ and CD27+ on NK cells subpopulations across follow-up in KS/HIV patients.

		NK^{dim} Cells			NK^{high} Cells		
		W_0	W_4	W_{12}	W_0	W_4	W_{12}
**$CD57^+$**	**CT**	10.7 (5.2-14.1)			15.2 (6.6-26.4)		
	**MT**	8.0 (4.7-16.9)			10.8 (7.4-37.0)		
**$CD27^+$**	**CT**	8.8 (4.8-11.5)			11.5 (8.6-13.3)		18.7 (17.1-25.0)
	**MT**	5.1 (3.4-7.7)	17.0 (14.9-21.6)		6.5 (4.6-14.5)	29.2 (20.8-31.5)	27.0 (22.6-32.2

Data are represented as median with interquartile range of the percentage. CT=conventional treatment (ART), MT= modified treatment (VGC+ART).

Table S5. PD-1 on the surface of circulating NK cells subsets.

		NK^{dim} Cells			NK^{high} Cells		
		W_0	W_4	W_{12}	W_0	W_4	W_{12}
**$CD57^+$**	**CT**	92.0 (29.0-109.2)	126.4 (49.0-161.0)	63.0 (57.5-116.4)	56.0 (42.0-100.0)	72.1 (44.3-153.2)	101.3 (89.0-119.0)
	**MT**	69.0 (44.5-76.1)	174.5 (94.9-246.5)	12.3 (6.7-119.8)	66.0 (46.2-82.0)	46.0 (34.1-64.0)	48.1 (18.0-75.0)
**$CD27^+$**	**CT**	160.2 (142.0-277.8)	240.0 (112.1-291.4)	72.4 (41.8-84.2)	584.5 (469.8-615.3)	261.5 (147.3-409.3)	163.5 (127.8-227.0)
	**MT**	238.0 (190.0-341.4)	210.3 (128.0-265.5)	203.0 (158.8-232.5)	591.0 (273.0-857.0)	558.5 (434.8-774.0)	458.5 (344.3-513.0)

Data are represented as median with interquartile range of the MFI. CT=conventional treatment (ART), MT= modified treatment (VGC+ART).

Table S6. KLRG1 on the surface of circulating NK cells subsets.

		NK^{dim} Cells			NK^{high} Cells		
		**W$_0$**	**W$_4$**	**W$_{12}$**	**W$_0$**	**W$_4$**	**W$_{12}$**
**CD57$^+$**	**CT**	43.2 (31.0-60.0)	56.0 (43.0-95.0)	58.0 (38.3-74.5)	25.0 (11.0-40.4)	54.3 (43.6-68.1)	69.4 (64.8-74.5)
	**MT**	48.8 (41.7-59.5)	55.0 (49.0-66.0)	61.3 (59.4-79.4)	5.6 (2.9-8.4)	38.8 (19.3-57.1)	67.0 (57.5-72.8)
**CD27$^+$**	**CT**	45.8 (29.5-59.1)	73.2 (52.6-147.3)	65.0 (57.0-86.0)	22.8 (17.9-37.5)	67.5 (53.9-76.9)	58.2 (49.1-73.8)
	**MT**	37.9 (30.9-47.1)	63.9 (49.8-80.7)	79.6 (53.6-93.6)	26.0 (19.8-31.6)	58.4 (41.4-80.0)	66.4 (55.0-68.7)

Data are represented as median with interquartile range of the MFI. CT=conventional treatment (ART), MT= modified treatment (VGC+ART).

Table S7. Frequency of NKT cells subsets in control groups included in this study.

**Subsets**	**HIV-Negative Men**	**HIV+ Asymptomatic Men**	***p***
NKT dim	67.2 (51.5 to 85.5)	85.9 (80.6 to 91.9)	ns
CD57+	44.0 (32.9 to 47.8)	48.5 (25.0 to 53.1)	ns
CD27+	8.22 (6.6 to 23.5)	14.2 (5.3 to 25.8)	ns
NKT high	34.9 (14.4 to 48.4)	14.1 (8.0 to 19.4)	ns
CD57+	44.8 (24.4 to 48.0)	57.2 (43.4 to 74.3)	ns
CD27+	9.1 (6.6 to 11.3)	6.8 (4.9 to 13.0)	ns

Data are represented as median with interquartile range. Statistical comparisons were performed using Mann–Whitney U test, CI 95%. (ns: not-significant). HIV, Human immunodeficiency virus 1.

Table S8. Frequencies of CD57+ and CD27+ on NKT cells subpopulations across follow-up in KS/HIV patients.

		NKT^{dim} Cells			NKT^{high} Cells		
		**W$_0$**	**W$_4$**	**W$_{12}$**	**W$_0$**	**W$_4$**	**W$_{12}$**
**CD57$^+$**	**CT**				27.7 (13.8-42.4)		
	**MT**				50.0 (44.9-68.7)	20.6 (10.2-34.6	24.2 (16.9-35.9)
**CD27$^+$**	**CT**	12.9 (7.5-22.1)	28.9 (20.0-41.5)		17.7 (14.2-24.8)		
	**MT**	6.7 (4.2-15.5)	19.7 (10.7-24.5)	18.7 (9.9-32.1)	13.1 (11.4-16.8)	33.3 (22.8-37.2)	25.5 (21.3-34.7)

Data are represented as median with interquartile range of the percentage. CT=conventional treatment (ART), MT= modified treatment (VGC+ART).

Table S9. PD-1 on the surface of circulating NKT cells subsets.

		NKT^dim Cells			NKT^high Cells		
		**W_0**	**W_4**	**W_{12}**	**W_0**	**W_4**	**W_{12}**
**CD57⁺**	**CT**	57.1 (29.0-120.0)	40.0 (33.0-89.0)	71.4 (39.0-99.0)	172.0 (118.5-259.5)	364.0 (250.5-402.0)	179.2 (162.5-277.1)
	**MT**	69.0 (50.0-96.0)	51.4 (34.3-116.0)	89.0 (48.0-160.0)	183.3 (106.2-292.0)	200.0 (129.2-262.5)	221.0 (123.0-279.2)
**CD27⁺**	**CT**	237.0 (178.5-338.3)	372.1 (300.0-428.5)	70.1 (55.5-90.9)	359.4 (271.0-490.0)	624.6 (211.3-924.0)	188.5 (82.9-324.3)
	**MT**	123.2 (104.5-337.5)	246.4 (171.4-396.4)	204.0 (123.2-241.1)	255.2 (203.1-755.2)	613.3 (558.8-759.0)	343.5 (166.3-64.5)

Data are represented as median with interquartile range. CT=conventional treatment (ART), MT= modified treatment (VGC+ART).

Table S10. KLRG1 on the surface of circulating NKT cells subsets.

		NKT^dim Cells			NKT^high Cells		
		**W_0**	**W_4**	**W_{12}**	**W_0**	**W_4**	**W_{12}**
**CD57⁺**	**CT**	62.5 (49.0-71.0)	71.5 (60.0-88.0)	69.4 (60.4-79.5)	40.4 (23.4-50.7)	52.0 (41.0-95.0)	64.6 (48.8-71.3)
	**MT**	65.2 (50.0-74.0)	74.3 (64.0-87.0)	64.0 (55.2-75.0)	61.0 (22.0-125.0)	56.0 (13.0-82.0)	68.0 (55.0-83.3)
**CD27⁺**	**CT**	52.0 (42.3-67.4)	58.3 (47.0-94.4)	71.0 (61.1-96.2)	36.8 (31.0-53.4)	86.1 (54.1-114.2)	188.5 (82.9-324.3)
	**MT**	53.2 (45.2-63.8)	68.1 (54.1-85.0)	75.8 (60.9-83.5)	51.0 (34.7-57.0)	73.2 (64.1-88.2)	343.5 (166.3-464.5)

Data are represented as median with interquartile range. CT=conventional treatment (ART), MT= modified treatment (VGC+ART).

Table S11. Plasma levels of PD-L1, PD-L2, E-cadherin, and IL-15.

	**HIV-Negative Men**	**HIV+ Asymptomatic Men**	**KS/HIV**			
Molecule/treatment			CT	*p*	MT	*p*
IL-15	9.04 (7.8-9.8)	10.2 (8.1-11.6)	18.60 (13.8-19.7	0.0005	18.3 (11.7-26.0)	0.0004
PD-L1	75.2 (48.5-135.5)	116.8 (73.1-459.0)	157.3 (138.0-468.0)	0.0173	156.0 (156.0-244.0)	0.0215
PD-L2	3112.0 (2159-4412)	7761.0 (4567.0-9183.0)	8961.0 (6784.0-10577.0)	0.0006	8081.0 (6397.0-10536.0)	0.0018
E-cadherin	302.6 (230.6-420.6)	448.0 (273.0-612.2)	990.0 (763.3-395.0)	0.0019	1030.0 (559.2-1857.0)	0.0023

Data are represented as median with interquartile range in pg/mL. Statistical comparisons were performed using the Kruskal-Wallis test, and the *p*-value represents the comparison between HIV-negative men and KS/HIV groups. HIV, Human immunodeficiency virus 1; CT=conventional treatment (ART), MT= modified treatment (VGC+ART).
